# Chronic erythema nodosum leprosum in Hansen’s disease: a challenging clinical management

**DOI:** 10.1093/trstmh/trag091

**Published:** 2026-08-22

**Authors:** Michele Spinicci, Anna Barbiero, Tommaso Manciulli, Costanza Fiorelli, Roberto Fedi, Alessandro Bussotti, Elena Silvestri, Danilo Malandrino, Giacomo Emmi, Giovanni Gazzoli, Lorenzo Zammarchi, Alessandro Bartoloni

**Affiliations:** Department of Experimental and Clinical Medicine, University of Florence, Largo Brambilla 3, 50134 Florence, Italy; Infectious and Tropical Diseases Unit, Careggi University Hospital, Largo Brambilla 3, 50134 Florence, Italy; Department of Experimental and Clinical Medicine, University of Florence, Largo Brambilla 3, 50134 Florence, Italy; Infectious and Tropical Diseases Unit, Careggi University Hospital, Largo Brambilla 3, 50134 Florence, Italy; Infectious and Tropical Diseases Unit, Careggi University Hospital, Largo Brambilla 3, 50134 Florence, Italy; Internal Medicine D Unit, Careggi University Hospital, Largo Brambilla 3, 50134 Florence, Italy; Casa Stenone, Fondazione Solidarità Caritas ETS, Via di Pulicciano 77, 50012 Bagno a Ripoli, Italy; Department of Experimental and Clinical Medicine, University of Florence, Largo Brambilla 3, 50134 Florence, Italy; Internal and Specialistic Medicine Unit, Careggi University Hospital, Largo Brambilla 3, 50134 Florence, Italy; Department of Experimental and Clinical Medicine, University of Florence, Largo Brambilla 3, 50134 Florence, Italy; Internal and Specialistic Medicine Unit, Careggi University Hospital, Largo Brambilla 3, 50134 Florence, Italy; Clinical Medicine and Rheumatology Unit, Cattinara University Hospital, Strada di Fiume 447, 34149 Trieste, Italy; Department of Medical, Surgery and Health Sciences, University of Trieste, Strada di Fiume 447, 34149 Trieste, Italy; Medical & Scientific Department, AIFO ETS, Via Girolamo Borselli 4-6, 40135 Bologna, Italy; Department of Experimental and Clinical Medicine, University of Florence, Largo Brambilla 3, 50134 Florence, Italy; Infectious and Tropical Diseases Unit, Careggi University Hospital, Largo Brambilla 3, 50134 Florence, Italy; Department of Experimental and Clinical Medicine, University of Florence, Largo Brambilla 3, 50134 Florence, Italy; Infectious and Tropical Diseases Unit, Careggi University Hospital, Largo Brambilla 3, 50134 Florence, Italy

**Keywords:** anti-TNF-α, clofazimine, ENL, leprosy, leprosy reaction, thalidomide, type II reaction

## Abstract

Introduction. Chronic erythema nodosum leprosum (ENL) is a challenging complication of Hansen’s disease (HD), with limited treatment options and frequent steroid dependence. Anti-tumour necrosis factor (TNF)-α agents are promising alternatives, though evidence remains confined to a few reports.

Case description. We report the case of a 34-year-old Moroccan man with multibacillary Hansen’s disease, who developed chronic ENL with recurrent systemic and cutaneous flares, requiring prolonged immunomodulatory therapy. Corticosteroids, thalidomide, azathioprine, methotrexate, and anti-TNF-α therapy only provided incomplete control. After six years of recurrences requiring several hospitalizations, treatment with methylprednisolone, high-dose thalidomide, and clofazimine achieved a sustained remission, allowing gradual treatment discontinuation.

## Case report

In May 2019, a 34-year-old man from Morocco was diagnosed with multibacillary Hansen’s disease (borderline lepromatous leprosy, according to the Ridley–Jopling classification) through *Mycobacterium leprae* detection on skin biopsy and multisite slit-skin smears, where a 3–4+ bacillary index was observed. Multidrug therapy (MDT) was started with rifampicin 600 mg once/month, dapsone 100 mg/day, and clofazimine 300 mg once/month, then 50 mg daily. Recurrent episodes of fever, arthralgia, lymphadenopathy and skin lesions were occurring for 2 years before Hansen’s disease diagnosis and continued after MDT introduction in association with polyneuropathy, being classified as erythema nodosum leprosum (ENL). Immunomodulatory therapy was therefore introduced starting from July 2019, initially with prednisone 50 mg/day, thalidomide 50–150 mg/day, later combined with methotrexate 7.5 mg/week, followed by cyclosporine 5 mg/kg/day, achieving only incomplete ENL control. In February 2020, a surgical intervention for ulnar nerve decompression was required. Furthermore, in December 2020, due to persistence of intact *M. leprae* bacilli on the bacilloscopic examination performed on skin biopsies and nasal mucosa, MDT was switched to rifampicin, minocycline 100 mg/day, and moxifloxacin 400 mg/day.

In May 2022, owing to ENL recurrences and the development of steroid-induced type 2 diabetes mellitus, anti-tumour necrosis factor (TNF)-α etanercept 25 mg twice/week was introduced, aiming to taper corticosteroid doses. In November 2022, etanercept was replaced with infliximab (5 mg/kg every 8 weeks) because of inadequate disease control.

In April 2023, bacilloscopy yielded negative results and MDT was discontinued after 48 months. Nevertheless, in the following 18 months the patient was hospitalized on multiple occasions due to severe ENL, characterized by febrile episodes associated with subcutaneous nodules, lymphadenopathy, and arthralgia (Fig. 1A). Acute manifestations were managed with high-dose corticosteroids, followed by gradual tapering. During this period, a comprehensive diagnostic work-up was performed to rule out potential triggers, such as chronic bacterial or parasitic infections, odontostomatological disorders, and peptic ulcer. However, flares continued even when the interval between infliximab infusions was shortened to 4 weeks, and the dose was incremented from 5 to 7 mg/kg. In January 2024, during an ENL flare, a skin biopsy was repeated and revealed a perivascular and periannexal dermatitis with a superficial and deep pattern with lympho-histiocytes, while the histochemical investigations did not demonstrate the presence of microorganisms. Moreover, serial electromyographies documented a sensory-motor polyneuropathy of moderate to severe degree, with consistently greater involvement of the left ulnar nerve. Considering the failure of anti-TNF-α treatment in quieting chronic ENL, the immunomodulatory regimen was switched to methylprednisolone 40 mg/day, thalidomide 300 mg/day, and clofazimine 300 mg/day in November 2024. Since the beginning of this combination, no ENL recurrence was observed, allowing a programme of extremely slow tapering to be started, which led to discontinuation of thalidomide and clofazimine in July 2025. Although the patient achieved a sustained clinical response, a high-resolution ultrasound of peripheral nerves performed in September 2025, while taking methylprednisolone 4 mg/day, evidenced signs of active left ulnar neuropathy (Fig. 1B). Low-dose steroid has been tapered further and then discontinued in March 2026. Approximately 1 month after steroid discontinuation, the patient developed secondary adrenal insufficiency, manifesting as myalgia, abdominal pain, anorexia, unintentional weight loss, postural hypotension, and profound fatigue, requiring glucocorticoid replacement therapy with cortisone acetate 25 mg/day.

**Figure 1:**
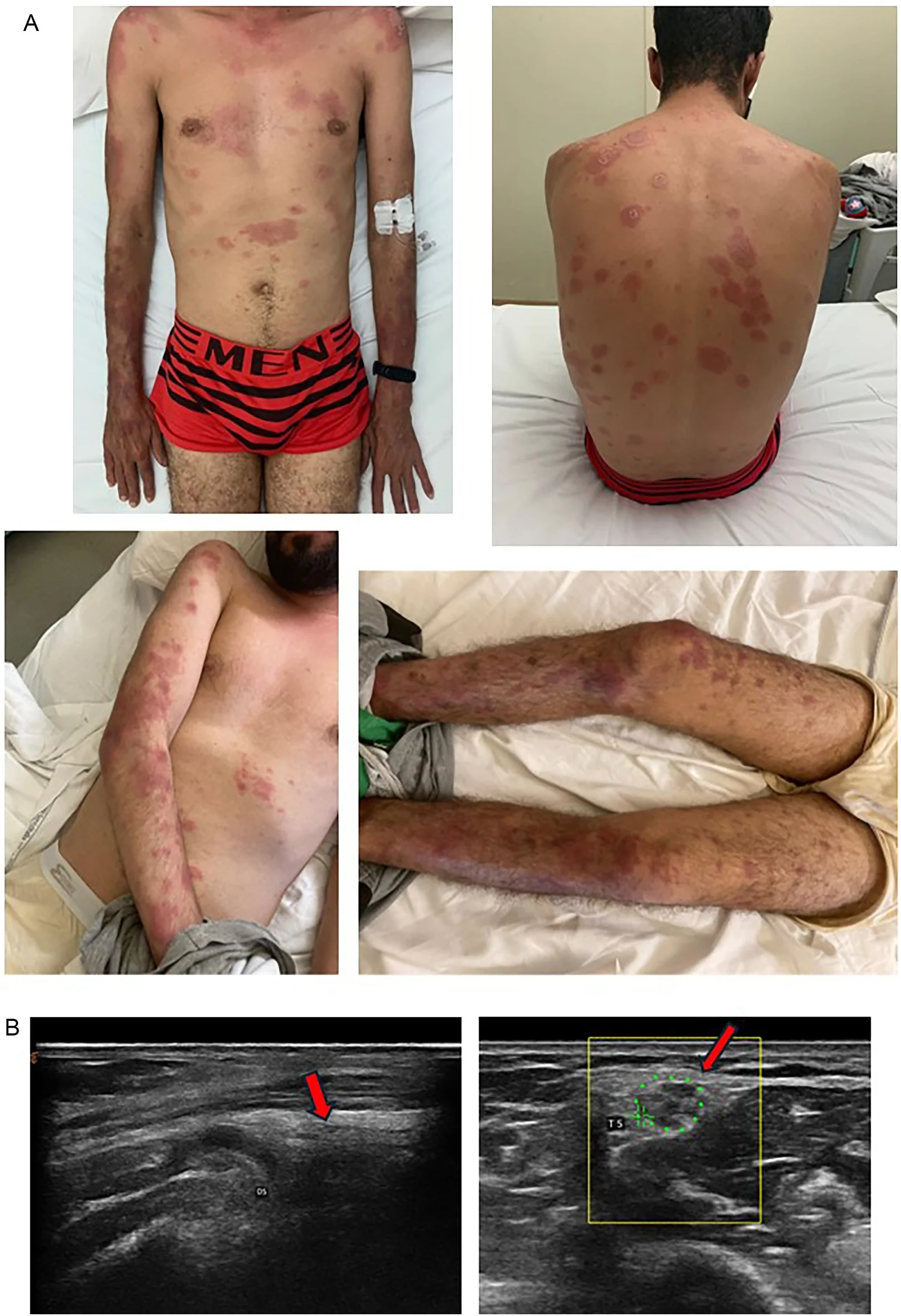
**(A)** Cutaneous manifestation during ENL flares. The lesions appear as erythematous, raised, painful nodules and plaques widespread across the body, including trunk, limbs, and face (not shown). Cutaneous findings were accompanied by systemic features including fever, arthralgia, and lymphadenopathy. **(B)** Signs of active left ulnar neuropathy evidenced by high-resolution ultrasound of peripheral nerves. Left: Longitudinal scan of the ulnar nerve at the posteromedial elbow region—the nerve appears thickened and hypoechoic (arrow). Right: Axial scan of the ulnar nerve at the posteromedial elbow region (arrow)—nerve echotexture appears irregular with hypoechoic areas. Cross-sectional area is increased at 14 mm², consistent with an active inflammatory process (reference values for ulnar nerve cross-sectional area at the cubital tunnel: 6.6 ± 1.5 mm²; Voltan G, *et al. Radiol Bras*. 2022;55(6):337–345).

A timeline summarizing the 6-year therapeutic course is reported in Fig. 2. A list with the details of all treatment regimens is provided in the Supplementary material (Table S1).

**Figure 2:**
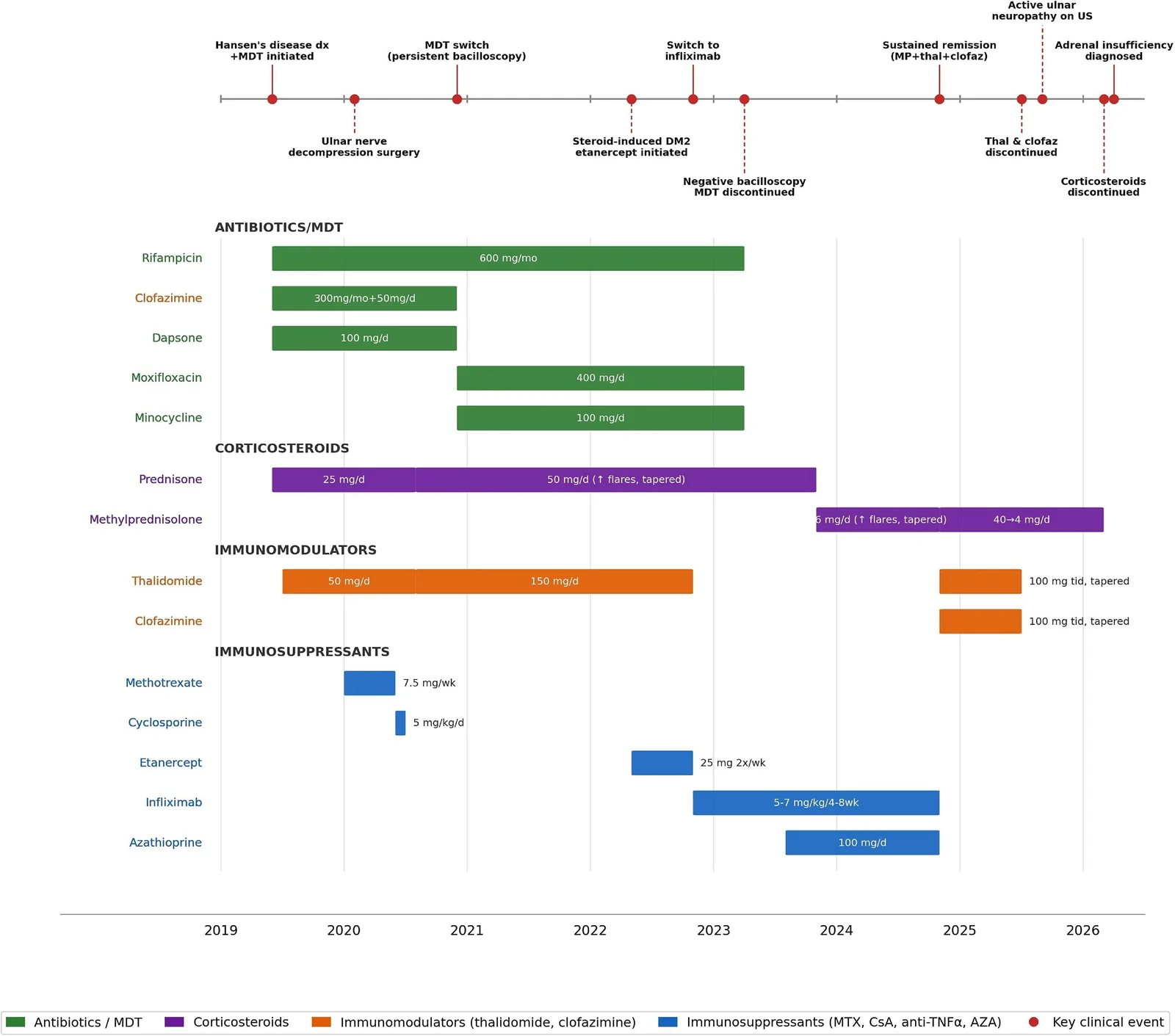
Clinical timeline of disease course and treatment in a patient with chronic erythema nodosum leprosum (ENL). Horizontal bars indicate treatment duration; dose annotations are shown inside or below each bar. Red dashed lines mark key clinical events. MDT = multidrug therapy; MTX = methotrexate; CsA = cyclosporine; AZA = azathioprine; anti-TNFα = tumour necrosis factor-α inhibitor; US = ultrasound; DM2 = type 2 diabetes mellitus; mo = month; d = day; wk = week; q8w = every 8 weeks; q4w = every 4 weeks.

## Discussion

Leprosy, or Hansen’s disease, is a chronic granulomatous infectious disease caused by *Mycobacterium leprae* and *Mycobacterium lepromatosis*, characterized by a complex and multifaceted immunopathogenesis.^1^ Leprosy reactions are acute inflammatory, immunologically driven episodes that complicate the chronic indolent course of the disease in 30%–50% of cases, and can occur prior to, during, or following the completion of MDT.^2^ In particular, ENL, also known as type II reaction, is a severe immune complex-mediated complication, characterized by systemic inflammation and significant morbidity, occurring in patients with lepromatous (LL) and borderline lepromatous (BL) leprosy, with a high bacillary load.^3^ Chronic ENL may keep on recurring over several years; however, duration and severity observed in our case after completing MDT are anecdotal. The differentiation between ENL recurrence and bacteriological relapse was based primarily on clinical grounds. Bacilloscopy, which had remained positive in December 2020 despite MDT, turned negative in April 2023, effectively ruling out active bacillary replication at the time of the subsequent ENL flares. Furthermore, relapse was considered unlikely throughout the disease course given the absence of new skin lesions suggestive of active bacterial multiplication—a hallmark feature of leprosy relapse. In contrast, the clinical picture was dominated by recurrent acute systemic inflammatory exacerbations, characterized by sudden-onset fever, malaise, and arthralgia, with rapid albeit transient response to corticosteroids, all consistent with ENL rather than relapse, which typically follows a slow and progressive course with gradual appearance of new lesions.

While ENL management mostly relies on old, poorly tolerated drugs and steroid dependence is frequent, the use of anti-TNF-α agents has been reported as a promising option for ENL when conventional therapies are not sufficient to control symptoms. However, evidence is confined to a few reports and small series.^4,5^ In our case, anti-TNF-α therapy did not provide substantial clinical benefit and may have contributed to the delayed clearance of *M. leprae*, thus requiring prolonged MDT. However, given the concurrent use of multiple immunosuppressive drugs, a causal role of anti-TNF-α therapy cannot be established, and this interpretation should be taken with caution. Moreover, in the setting of persistent *M. leprae* detection, the choice of second-line antimycobacterial regimens should ideally be guided by drug susceptibility testing (DST). The unavailability of DST represents a limitation of this case report. ENL remission was achieved through a multidrug regimen consisting of high-dose thalidomide (300 mg/day), clofazimine at anti-inflammatory dose (300 mg/day), and an extremely gradual tapering of corticosteroids, in accordance with evidence reported in the WHO guidance.^2^ The relative contribution of each individual component of this combination cannot be precisely determined. In addition, a spontaneous attenuation of ENL activity over time—as may occur in the natural history of the disease—cannot be excluded as a contributing factor to the observed clinical outcome. Other drugs have been used as second-line treatments for ENL in small-scale studies, including methotrexate, pentoxifylline, and apremilast, which is an oral phosphodiesterase-4 inhibitor sharing a common molecular structure with thalidomide.^6–8^

## Conclusions

This case highlights the substantial challenges associated with the management of leprosy reactions. Improved understanding of the complex interplay between *M. leprae* and the host immune system may inform more effective strategies for the treatment and prevention of leprosy reactions. Furthermore, the impact of immunosuppressive therapies, including anti-TNF-α agents, on the natural history of Hansen’s disease warrants further investigation. In this context, the expanding global use of immunosuppressive drugs, particularly in regions where Hansen’s disease remains endemic, requires careful monitoring.

## Supplementary Material

trag091_Supplemental_File

## Data Availability

The data supporting the findings of this case report are available from the corresponding author upon reasonable request.
