# Supplementary material for: Chronic erythema nodosum leprosum in Hansen’s disease: a challenging clinical management
Source: Trans R Soc Trop Med Hyg. 2026 Aug 22;120(11):1174–8. doi: 10.1093/trstmh/trag091 (PMC13728645; doi:10.1093/trstmh/trag091)
Supplement: Supplementary file 1 [file trag091_supplemental_file.docx]

**Tabler S1. Summary of treatments administered between June 2019 and June 2026**

| **Drug** | **Dose** | **Period** |
| --- | --- | --- |
| **Multidrug therapy** | | |
| Rifampin | 600mg once a month | June 2019 – April 2023 |
| Clofazimin | 300mg once a month, 50 mg daily | June 2019 – December 2020 |
| Dapsone | 100mg / day | June 2019 – December 2020 |
| Moxifloxacin | 400mg/day | December 2020 – April 2023 |
| Minocycline | 100mg/day | December 2020 – April 2023 |
| **Immunosuppressants** | | |
| Prednisone | 25 mg/day | June 2019 – August 2020 |
|  | 50 mg/day (high doses during ENL flare then tapered) | August 2020 – November 2023 |
| 6-Methylpredinsolon | 16mg / day (high doses during ENL flare then tapered) | November 2023 – November 2024 |
| Metothrexate | 7.5mg/week | January 2020 - June 2020 |
| Cyclosporine | 5 mg/kg/day in two doses | June 2020 – July 2020 |
| Thalidomide | 50mg / day | July 2019 – August 2020 |
|  | 150 mg/day | August 2020 – November 2022 |
| Etarnecept | 25 mg twice a week | May 2022 – November 2022 |
| Infliximab | 5 mg/kg every 8 weeks | November 2022 – September 2023 |
|  | 7 mg/kg every 4 weeks | September 2023 – December 2023 |
|  | 5 mg/kg every 4 weeks | December 2023 – November 2024 |
| Azathioprine | 100 mg / day | August 2023 – November 2024 |
| **Last regimen** | | |
| 6-Methylpredinsolon | 40 mg/day gradually tapered until 4mg twice/week | November 2024 – March 2026 |
| Thalidomide | 100 mg three times/day | November 2024 – December 2024 |
|  | 100 mg two times/day | December 2024 - March 2025 |
|  | 100 mg once /day | March 2025 – July 2025 |
| Clofazimin | 100 mg three times / day | November 2024 – December 2024 |
|  | 100 mg two times / day | December 2024 – March 2025 |
|  | 100 mg once / day | March 2025 – July 2025 |
